# Exposure to structurally unique β‐d‐glucans differentially affects inflammatory responses in male mouse lungs

**DOI:** 10.14814/phy2.16115

**Published:** 2024-06-23

**Authors:** Nervana Metwali, Emma M. Stapleton, Suzana Hadina, Peter S. Thorne

**Affiliations:** ^1^ Department of Occupational and Environmental Health College of Public Health, University of Iowa Iowa City Iowa USA; ^2^ Division of Pulmonary Critical Care and Occupational Medicine, Department of Internal Medicine University of Iowa Iowa City Iowa USA; ^3^ Present address: Department of Microbiology & Infectious Disease with Clinic Faculty of Veterinary Medicine, University of Zagreb Zagreb Croatia

**Keywords:** Beta‐glucan, immunology, inhalation exposure, murine, pustulan

## Abstract

Pro‐inflammatory fungal β‐d‐glucan (BDG) polysaccharides cause respiratory pathology. However, specific immunological effects of unique BDG structures on pulmonary inflammation are understudied. We characterized the effect of four unique fungal BDGs with unique branching patterns, solubility, and molecular weights in murine airways. Scleroglucan (1 → 3)(1 → 6)‐highly branched BDG, laminarin (1 → 3)(1 → 6)‐branched BDG, curdlan (1 → 3)‐linear BDG, and pustulan (1 → 6)‐linear BDG were assessed by nuclear magnetic resonance spectroscopy. Each BDG was tested by inhalation model with C3HeB/FeJ mice and compared to saline‐exposed control mice and unexposed sentinels (*n* = 3–19). Studies were performed ±heat‐inactivation (1 h autoclave) to increase BDG solubility. Outcomes included bronchoalveolar lavage (BAL) differential cell counts (macrophages, neutrophils, lymphocytes, eosinophils), cytokines, serum IgE, and IgG2a (multiplex and ELISA). Ex vivo primary cells removed from lungs and plated at monolayer were stimulated (BDG, lipopolysaccharide (LPS), anti‐CD3), and cytokines compared to unstimulated cells. Right lung histology was performed. Inhalation of BDGs with distinct branching patterns exhibited varying inflammatory potency and immunogenicity. Lichen‐derived (1 → 6)‐linear pustulan was the most pro‐inflammatory BDG, increasing inflammatory infiltrate (BAL), serum IgE and IgG2a, and cytokine production. Primed lung cells responded to secondary LPS stimulation with a T‐cell‐specific response to pustulan. Glucan source and solubility should be considered in exposure and toxicological studies.

## 1 INTRODUCTION

Damp and moldy environments are known contributors to respiratory pathology, including allergic asthma, in children and adults (Andrae et al., 1988; Cuijpers et al., 1995; Laney et al., 2009; Waegemaekers et al., 1989). For example, increased exposure to fungal cell wall material, β‐d‐glucan (BDG), is negatively associated with FEV_1_ and increases the risk of emergency/urgent care visits in asthmatics by nearly nine‐fold in children (Blatter et al., 2014). Exposure to indoor bacterial endotoxin and mold BDG are known respiratory irritants and potent cytokine inducers (Cavaillon, 2018; Douwes et al., 2000; Kizhakke Puliyakote et al., 2023).

β‐d‐glucan structures vary depending on their source and extraction process (Bohn & BeMiller, 1995; Kollár et al., 1997). (1 → 3)‐β‐d‐glucans (1,3‐BDGs) are major structural components of fungal cell walls that activate macrophages and modulate innate immunity. Microorganism‐derived BDGs generally have (1 → 3)‐linked anhydro‐d‐glucose units as a backbone with periodic (1 → 6)‐linked side chains. Other glucans have linear (1 → 3) linkage without branching. Numerous studies have revealed critical BDG properties include their molecular weight (MW) and water solubility, which depend on the degree of branching (DB) (Suzuki et al., 1991; Williams et al., 1991, 1992). Glucans are typically most potent between 100 and 200 kDa with the DB ranging from 0.2 to 0.5 (Bohn & BeMiller, 1995). Various destructive and nondestructive methods are available to define BDG molecular structure, DB, and degree of polymerization. Non‐destructive light‐scattering techniques are sensitive to macromolecular aggregation and provide glucan nuclear magnetic resonance spectroscopy (NMR) and polydispersity (Williams et al., 1991).

Glucan properties have downstream immunomodulatory effects. For example, BDG receptor Dectin‐1, a non‐toll‐like receptor (TLR) pattern‐recognition receptor (PRR), uniquely recognizes soluble and particulate glucans; soluble glucans attenuate Dectin‐1 signaling (Brown, 2006; Goodridge et al., 2011). BDG properties can be exploited therapeutically as biological‐response modifiers to improve host immunity (De Marco et al., 2021; Novakovic et al., 2016; Stothers et al., 2021). For example, branched 1,3‐BDGs including lentinan (Chihara et al., 1969), schizophyllan (Rau & Parsegian, 1990), and krestin (Mizutani et al., 1992) provide antitumor activity in the host. Soluble glucans are also thought to have unique anti‐tumor effects (Wu et al., 2021). Yeast cell wall‐derived zymosan is a potent macrophage activator and neutrophil chemoattractant, binding to membrane components such as scavenger receptor complement receptor 3, Lactosylceramide, and Dectin‐1 with TLR2 and TLR6 heterodimer signaling (Brown et al., 2002; Reichner et al., 2001; Sorenson et al., 1998; Underhill, 2003). Linear 1,3‐BDGs (e.g., curdlan) can also stimulate macrophage binding to pattern‐recognition receptors using MyD88 for signal transduction (Kataoka et al., 2002).

Specific 1,3‐BDG physicochemical parameters, including primary structure, solution conformation, MW, and/or polymer charge are thought to affect macrophage receptor affinity. However, this relationship has not been defined, in part due to a lack of well‐characterized BDG polymers with varying MWs and conformations. Their effect on respiratory inflammation is understudied. Therefore, we evaluated the effect of four glucan compounds on lung inflammation using an in vivo murine allergen inhalation model.

## 2 MATERIALS AND METHODS

Catalog numbers listed in Data S1.

### 2.1 Glucan compounds

Scleroglucan (1 → 3)(1 → 6)‐highly branched BDG was generously provided by Dr. David Williams (Tennessee State University). Commercial suppliers provided laminarin ((1 → 3)(1 → 6)‐branched BDG; Sigma‐Aldrich, Inc., St. Louis, MO), curdlan ((1 → 3)‐linear BDG; Wako, Richmond, VA), and pustulan ((1 → 6)‐linear BDG; Calbiochem Inc., La Jolla, CA). Glucan compounds were confirmed negative for endotoxin by kinetic chromogenic Limulus amebocyte lysate assay.

### 2.2 Determination of the degree of glucan branching

The degree of branching was determined by nuclear magnetic resonance (NMR). NMR experiments were performed using a Bruker FT‐NMR spectrometer DMX 600 resonating at 600.13 MHz for 1H at the University of Iowa NMR facility core. Glucan compounds were dissolved in deuterium oxide (D2O) or mixtures of PBS‐D2O. Two‐dimensional magnitude correlation spectroscopy (COSY) experiments were performed to assign the 1H resonance.

### 2.3 Animal model

Four‐ to five‐week‐old, pathogen‐free male C3HeB/FeJ mice were obtained from Jackson Laboratories (Bar Harbor, ME) and housed in a rodent vivarium with a 12 h light–dark cycle and provided with food (Formula 7913, NIH‐31 Irradiated Modified Open Formula Mouse/Rat Diet, Inotive, West Lafayette, IN), and water ad libitum. The C3HeB/FeJ strain was selected to evaluate the inflammatory response to fungal cell wall material given previous work demonstrating these mice exhibit robust inflammatory response to environmental bioaerosol exposure (George et al., 2006; Kulhankova et al., 2009; Mueller‐Anneling et al., 2006). Mice were quarantined for 10 days prior to BDG glucan exposure. All animal protocols were reviewed and approved by the Office of the Institutional Animal Care and Use Committee at the University of Iowa, #0407150.

### 2.4 β‐d‐glucan conjugation

We performed BDG conjugation using previously described methods (Douwes et al., 1996; Meikle et al., 1991; Montijn et al., 1994; Roy et al., 1984). Curdlan and pustulan were oxidized using 2 mL of 20 mg/mL in pyrogen‐free water (pH 6.5) using 2 mL 0.5 M NaIO_4_, incubated (20°C, 60 min), and the reaction stopped with 250 μL ethylene glycol. The solution was desalted (6000 MW exclusion column equilibrated with 0.2 M borate buffer, pH 9), 1 mL was collected, and carbohydrate concentration was measured per carbohydrate concentration kit instructions (Thermo Fisher Scientific, Waltham, MA). BDG conjugation fractions were pooled into three groups determined by column arrival time, representing different chain lengths for a final ratio of 1:0.5:1 (glucan, bovine serum albumin (BSA), Na cyanoborohydride). After the solution was agitated (5 days in water bath, 40°C), BDGs were separated on 6000 MW exclusion column equilibrated with 20 mM PBS pH 7.4. The heaviest carbohydrate (phenol‐sulfuric acid) and protein (OD280) fractions were pooled and concentrated with a centriplus concentrator (Thermo Fisher Scientific), re‐separated, and combined with the heaviest BSA. The final injection mixture contained equal volumes of three conjugate mixes. Branched glucans (scleroglucan, laminarin) were treated identically, except 2 mL of CF₃CO₂H (0.2 M) was used for hydrolyzation.

### 2.5 β‐d‐glucan murine exposure

On days 0 and 7, mice received intraperitoneal (i.p.) injections of glucan‐bovine serum albumin conjugates (glucan‐BSA) with an adjuvant of glucan (25 μg/mL) emulsified with 1 mg/mL aluminum hydroxide (alum) suspended in saline. Negative control mice were exposed to saline. On days 14–16, 21–23, and 28–30, mice previously exposed to BDGs were challenged intranasally with 25 μg BDG/mouse, suspended in 50 μL saline, while control mice received saline solution (Figure S1). A sub‐study was performed using BDGs autoclaved for 1 h prior to nasal instillation. Procedures were performed under anesthesia, the dose of which was determined by the loss of reaction to a pinch. Euthanasia (i.p. pentobarbital, 150 mg/kg) and exsanguination were performed on day 31.

### 2.6 Bronchoalveolar lavage fluid collection and analysis

Lungs were lavaged with sterile, pyrogen‐free saline at a pressure of 25 cmH_2_O in 1 mL increments for a total volume of 4 mL. BAL was stored at 4°C and processed as soon as possible after collection (cell‐type quantification preformed within 24 h of collection, protein analysis performed within 7 days after storage in −80°C freezer). The BAL was centrifuged for 5 min at 800 × *g*, and the resulting supernatant was decanted, divided into equal volume aliquots. BAL was analyzed by multiplex immunoassay bead array systems (BioPlex, Bio‐Rad, Hercules, CA) per manufacturer instructions. BAL was analyzed for IL‐6, IL‐9, IL‐10, IL‐12p40, IL‐17, CXCL‐1, MCP‐1/CCL‐2, MIP‐1α/CCL‐3, MIP‐1β/CCL‐4, and Eotaxin. The limit of detection was 10 pg/mL for each assay. Sandwich enzyme‐linked immunoassays (ELISAs) were performed using paired antibodies (eBioscience, Invitrogen, Waltham, MA) for IFN‐γ, TGF‐β, and TNF‐α. The limit of detection was 10 pg/mL for each assay. Immune cell infiltrate in BAL was assessed using previously described cell counting methods (Schwartz et al., 1994).

### 2.7 Bronchoalveolar lavage fluid protein concentration and cell‐type

The BAL cell pellet, collected and processed on day 31, was re‐suspended in Hank's Balanced Salt Solution media with phenol red and prepared for total and differential cell counts. Total counts were performed using an improved Neubauer hemocytometer (Reichert, Buffalo, NY, USA), and the Diff Quick Stain Set (Thermo Scientific, USA) was used for staining for differential enumeration by microscopy. Remaining BAL was stored in a − 80°C freezer and total protein concentration analyzed within 7 days, determined using the QuantiPro BCA assay kit (Sigma‐Aldrich) by first interpolating the calibration curve using BSA standard solution (0.5–30 μg/mL) and concentration determined per manufacturer instructions. Briefly, 150 μL was added to the plate and incubated for 16 h, and absorbance was measured at 562 nm (SpectraMax Plus 384, Molecular Devices, Inc.). BAL was also analyzed for water‐soluble proteins.

### 2.8 Response of left lung lobe primary cells to secondary stimuli

Lungs were removed, then left lung lobes were manually syringed with media (repeat suction/expulsion cycles through 1 mL syringe RPMI 1640 (GIBCO, Grand Island, NY)) and cells dispersed, then passed through a 100 μm nylon cell strainer (BD Labware, Franklin, NJ). Red blood cells were lysed by hypotonic shock. Remaining cells were washed twice and re‐suspended in RPMI 1640 containing 10% FCS, 25 mM HEPES buffer, 2 mM l‐glutamine, 5 × 10^−5^ M β‐mercaptoethanol, 1 mM sodium pyruvate, 100 U/mL penicillin, and 100 mg/mL streptomycin (GIBCO) alongside vehicle or stimulation (BDG (10 μg/mL glucan), LPS (8 μg/mL derived from *Escherichia coli* 0111:B4, Sigma‐Aldrich), or with anti‐CD3 mAb (clone #2C11, ATCC) (1 μg/mL), which stimulates and activates T‐cells). Cells were plated in triplicate (96‐well microtiter plates, Corning, Cambridge, MA) (2 × 10^6^ cells/well) at 37°C and 5% CO_2_. After 48 h, the plate was centrifuged (800 × *g*, 10 min) and supernatant collected and stored at −80°C for cytokines analyses. Cytokines and chemokines evaluated included IL‐4, IL‐6, IL‐10, IL‐12/23p40, IL‐17, and MIP‐1α by multiplex immunoassay (Bio‐Rad, Hercules, CA).

### 2.9 Histopathology

Lungs of mice from each exposure group were fixed with 10% formaldehyde, embedded in paraffin, sectioned (4 μm thick), and stained with hematoxylin and eosin stain. The slides were evaluated for visual abnormalities and scored for inflammation.

### 2.10 Serum

Animals were anesthetized, euthanized, and exsanguinated by cardiac puncture to obtain whole blood. Blood from all animals of an exposure group was pooled and centrifuged (600 × *g*), and serum was isolated and assessed for immunoglobulin E (IgE) and IgG2a using sandwich ELISA (Invitrogen, Waltham, MA) according to manufacturer instructions.

### 2.11 Statistics

Statistical analyses were carried out using GraphPad Prism, version 10.0.0. Values below the limit of detection were divided by √2, and cell‐count values of 0 were changed to 0.5. Normality was assessed by Kolmogorov–Smirnov test. Within a given experiment, if data were normally distributed, one‐way ANOVAs were performed with Šídák's multiple comparisons test comparing control to each condition. If data were not normally distributed, a Kruskal‐Wallis test was performed with Dunn's multiple comparisons test. The number of BAL immune cells per mouse was compared to the number of cells per mL by linear regression. Sentinels, saline‐exposed and saline+alum‐exposed mice were compared by one‐way ANOVAs or Kruskal‐Wallis test with multiple comparisons. Figures denote *p* values ≤0.1 and significance was determined at *p* < 0.05.

## 3 RESULTS

### 3.1 Glucan structure

Glucan linearity was assessed by nuclear magnetic resonance (NMR, Figure S2). Each glucan compound used in this study has a unique branching pattern and chemical structure (Figure S3). Glucan source, molecular weight, and solubility characteristics can be found in Table 1.

**TABLE 1:** Structural characteristics and solubility of glucan compounds.

Name	Linkage	Source	Molecular weight	Linearity	Solubility in saline
Scleroglucan	(1➔6)(1➔3)‐β‐d‐glucan	*Sclerotium glucanincum*	3,300,000	Branched	Soluble
Laminarin	(1➔6)(1➔3)‐β‐d‐glucan	*Laminaria digitata*	58,500	Linear with side branching	Soluble
Curdlan	(1➔3)‐β‐d‐glucan	*Alcaligens faecalis*	136,000	Linear	Insoluble
Pustulan	(1➔6)‐β‐d‐glucan	*Umbiliccaria papullosa*	20,000	Linear	Soluble

*Note*: Molecular weight provided by BDG vendor. Linearity was assessed by NMR and solubility determined by visual assessment.

### 3.2 Global effects of β‐d‐glucan exposures

Mice were sensitized by i.p. injections to glucan conjugate emulsified with alum in saline. After 2 weeks, animals were challenged intranasally with individual glucans for a total of nine doses over 2 weeks, and protein concentration and cell‐type composition of BAL were analyzed. Saline‐exposed animals were used as a control unless otherwise indicated, given no significant differences were observed between saline and the saline+alum conditions (Figure S4).

**FIGURE 1:**
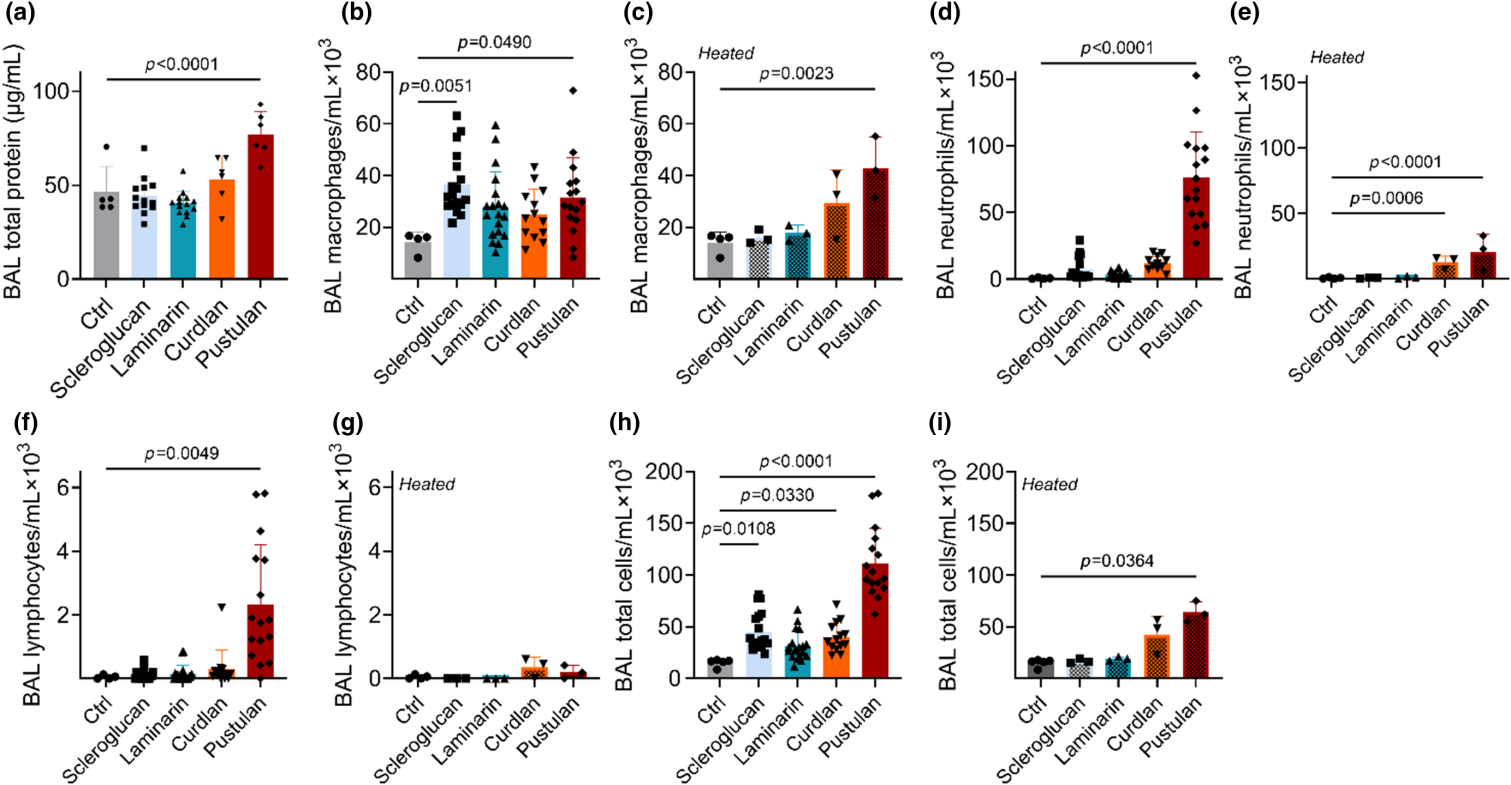
Total protein concentration and inflammatory cell infiltrate in bronchoalveolar lavage by glucan exposure ± heating. (a) BAL was collected on day 31 and total protein concentration assessed after exposure to each BDG; (b–g) Cell concentrations in BAL after exposure to the indicated BDG ± heating; BAL was collected on day 31 and cell‐types quantified immediately. (b, c) Macrophages; (d, e) Neutrophils; (f, g) Lymphocytes; (h, i) Total cells; significance determined by comparing control to each condition by one‐way ANOVA with Šídák's multiple comparisons test (total protein, macrophages, PMNs) or by Kruskal–Wallis test with Dunn's multiple comparisons test (lymphocytes, total cells); *n* = 3–19.

Exposure to pustulan, but no other glucan, led to the greatest BAL total protein concentration, Figure 1a. Analysis of the composition of BAL cells after exposure to each glucan revealed that pustulan induced the greatest influx of inflammatory cells, significantly increasing macrophage, neutrophil, lymphocyte, and total cell concentrations compared to controls (saline exposure), Figure 1b,d,f,h. Scleroglucan significantly increased macrophage and total cell concentrations while curdlan increased total cell concentration in BAL compared to controls, Figure 1b,h. Laminarin exposure was no different than controls for any of these outcomes.

**FIGURE 2:**
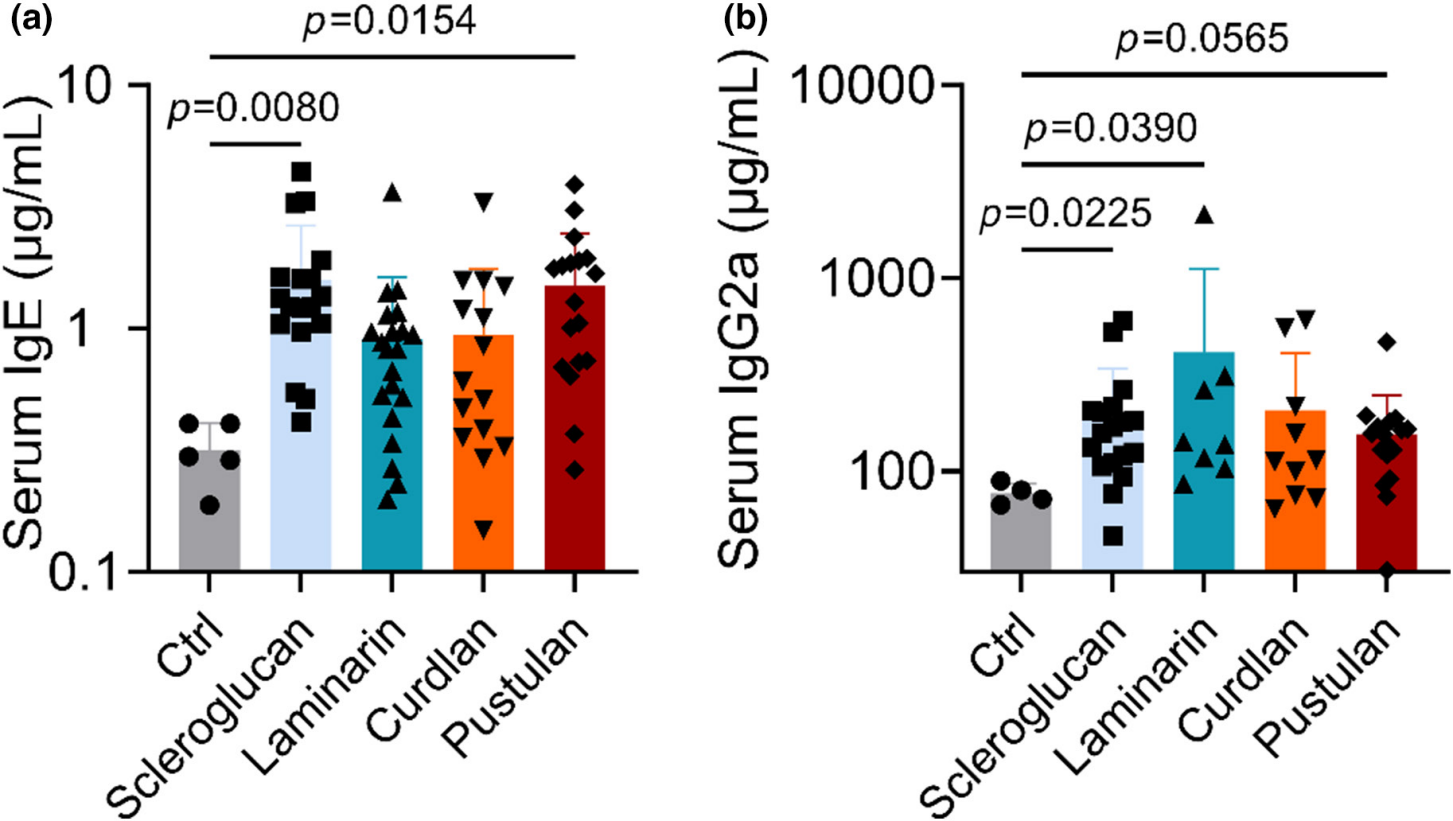
Effect of BDG exposure on serum IgE and IgG2a concentrations. (a) IgE concentration after exposure to each BDG. (b) IgG2a concentration after exposure to each BDG; significance determined by one‐way ANOVA with Šídák's multiple comparisons test (IgE) or by Kruskal–Wallis test with Dunn's multiple comparisons test (IgG2a); *n* = 4–21.

We then tested whether glucan structure differentially affected antibody production by assessing serum immunoglobulin (IgE and IgG2a) concentrations after exposure to individual glucans. Both IgE and IgG2a concentrations were elevated after exposure to scleroglucan and pustulan, while laminarin increased IgG2a concentrations compared to saline exposures (Figure 2a,b). Curdlan elevated both IgE and IgG2a; however, responses were variable and did not reach significance.

**FIGURE 3:**
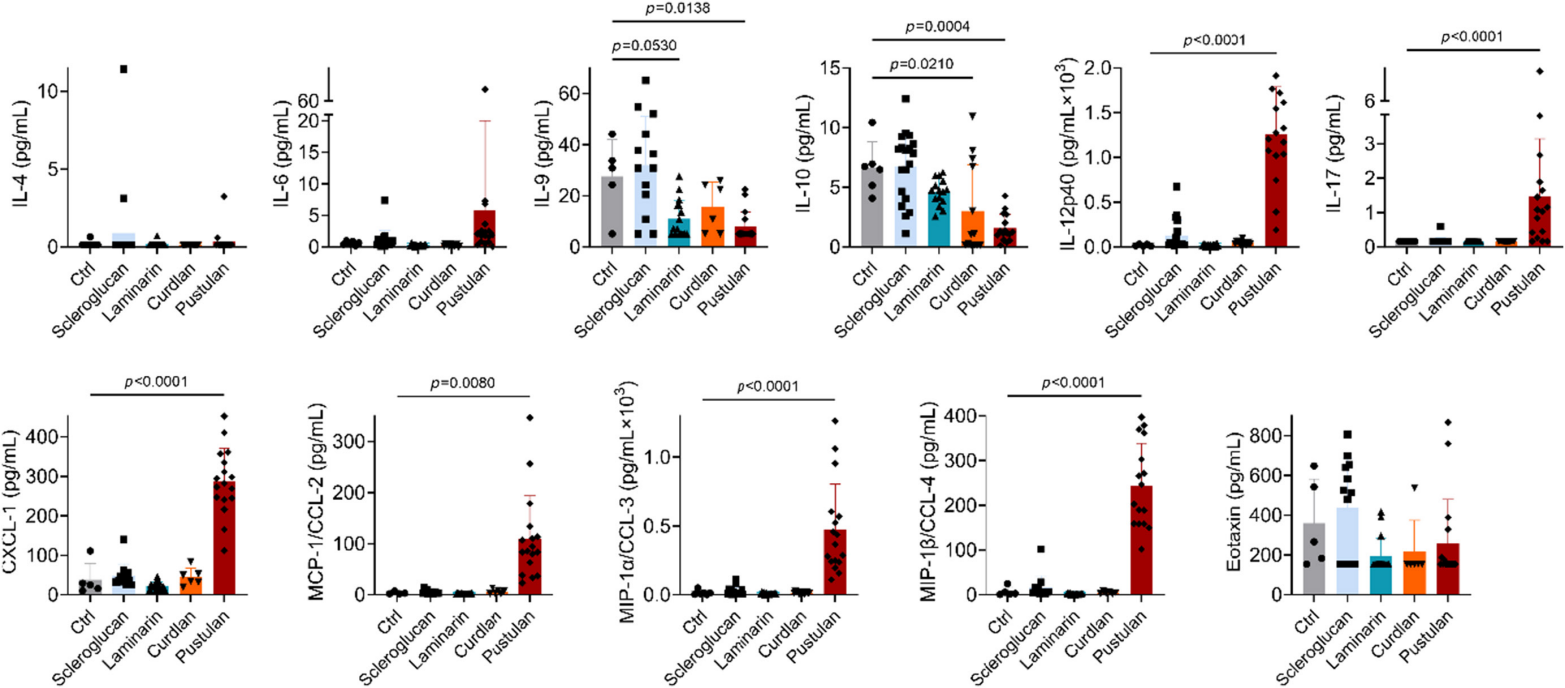
Cytokine concentration in bronchoalveolar lavage by BDG exposure. IL‐4, IL‐6, IL‐9, IL‐10, IL‐12p40, IL‐17, CXCL‐1, MCP‐1/CCL‐2, MIP‐1α/CCL‐3, MIP‐1β/CCL‐4 and Eotaxin concentration in BAL; significance determined by comparing control to each condition by one‐way ANOVA with Šídák's multiple comparisons test or by Kruskal–Wallis test with Dunn's multiple comparisons test depending on Kolmogorov–Smirnov test results; *n* = 5–19.

We analyzed BAL for the presence of Th1, Th2, and Th17 lineage cytokines to test whether exposure to unique glucan structures differentially affects cytokine production in the lungs. Pustulan induced the greatest number of changes in cytokine concentrations. Compared to saline control, pustulan increased IL‐12p40, IL‐17, CXCL‐1, CCL‐2, CCL‐3, CCL‐4 and decreased IL‐9 and IL‐10 production. Laminarin also decreased IL‐9 production, while curdlan decreased IL‐10 production, Figure 3. IL‐9 and IL‐10 both have anti‐inflammatory properties (Donninelli et al., 2020; Fang & Zhu, 2020; Goswami & Kaplan, 2011; Herfarth et al., 1996; Rauber et al., 2017). No changes to IFN‐γ, TGF‐β, and TNF‐α (tested by ELISA) were observed (Figure S5).

### 3.3 Effect of β‐d‐glucan solubility on murine lungs

Indoor BDG exposure quantification often relies on first heating dust samples prior to analysis (Choi et al., 2014; Rao et al., 2007; Yang & Huang, 2021), which increases their solubility. Soluble BDG is a Dectin‐1 antagonist, while particulate BDG is a Dectin‐1 agonist. Soluble BDGs might, however, agonize other non‐dectin‐1 β‐glucan PRRs (Borriello et al., 2022; Brown, 2006; Goodridge et al., 2011). Therefore, we sought to analyze the effect of solubility on lung inflammation. We performed a sub‐study on the effect of each compound after it was autoclaved (1 h) to assess the pro‐inflammatory effect. Overall, heating of BDGs led to less inflammatory infiltrate compared to unheated glucans (Figure 1). Within the heated sub‐study, curdlan and pustulan heating markedly increased recruitment of inflammatory cells to the lung, Figure 1c,e,i. Contrary to prior findings, heated scleroglucan no longer increased macrophage or total cell concentrations in BAL (Figure 1c,i). Although lymphocyte response was quite variable, heating of pustulan decreased the migration of lymphocytes into BAL by approximately one order of magnitude (Figure 1f,g).

**FIGURE 4:**
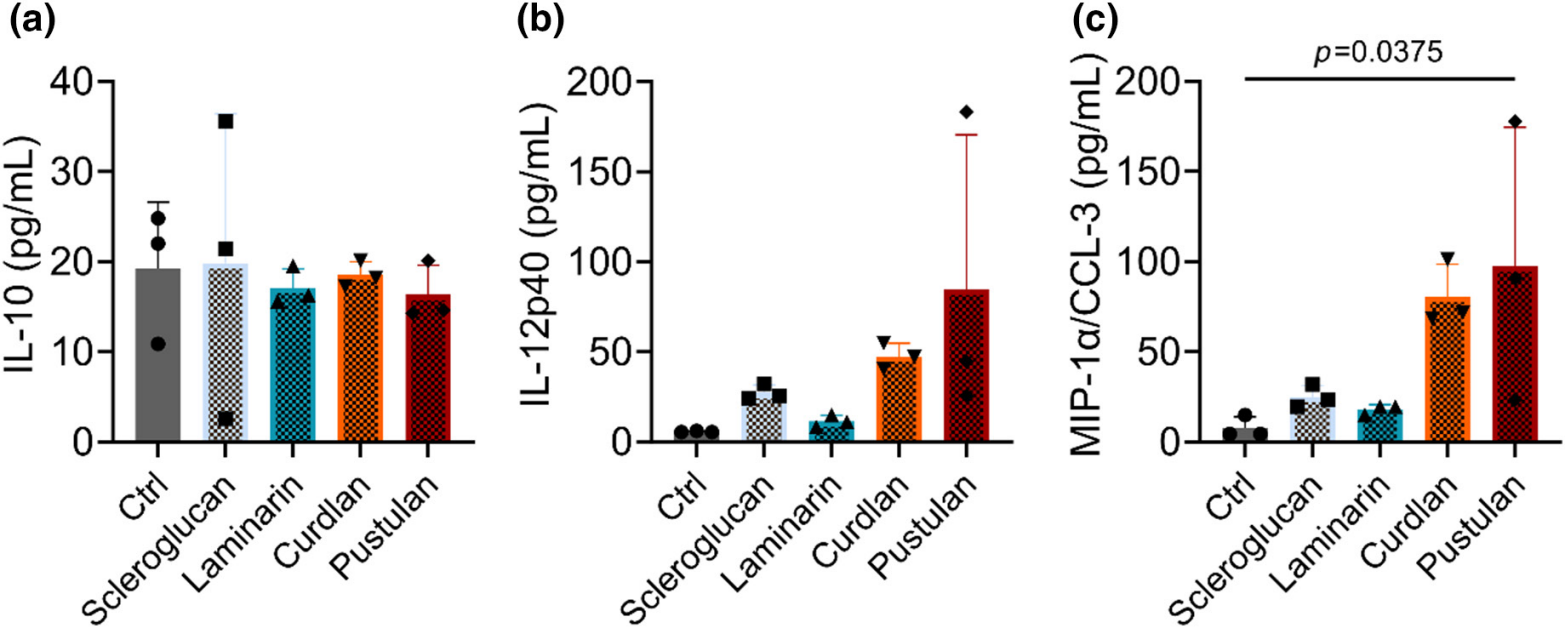
Effect of heating on cytokine concentration in bronchoalveolar lavage by glucan exposure. (a) IL‐10; (b) IL‐12p40; (c) MIP‐1α/CCL‐3 concentration in BAL; significance determined by comparing sentinel controls to each condition by one‐way ANOVA with Dunnett's multiple comparisons test; *n* = 3 per condition.

Next, we assessed the effect of glucan solubility on BAL cytokine expression. While cytokines were typically below the limit of detection, IL‐10, IL‐12p40, and CCL‐3 concentrations were detectable in BAL (Figure 4). Heated pustulan increased CCL‐3 concentration significantly compared to control (Figure 4c). Heating of glucans had no effect on TNF‐α (by ELISA) (Figure S5B).

**FIGURE 5:**
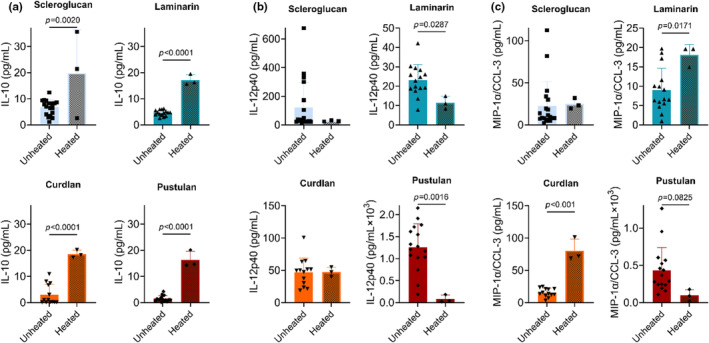
Effect of glucan heating on cytokine concentration in bronchoalveolar lavage by BDG exposure. (a) IL‐10 concentration in BAL. (b) IL‐12p40 concentration in BAL. (c) MIP‐1α/CCL‐3 concentration in BAL; significance determined by unpaired *t*‐test; *n* = 3–19.

We further tested the effect of glucan solubility on BAL cytokine concentrations by comparing unheated and heated glucans for IL‐10, IL‐12p40, and MIP‐1α/CCL‐3, Figure 5. Typically, heating of BDGs increased the concentrations of cytokines present in BAL. Heating of each glucan significantly increased the concentration of IL‐10, Figure 5a, while heated laminarin and pustulan decreased IL‐12p40, Figure 5b. We observed differential effects of BDG heating on MIP‐1α/CCL‐3. Heated laminarin and curdlan increased MIP‐1α/CCL‐3, while pustulan decreased MIP‐1α/CCL‐3 (trend), Figure 5c.

**FIGURE 6:**
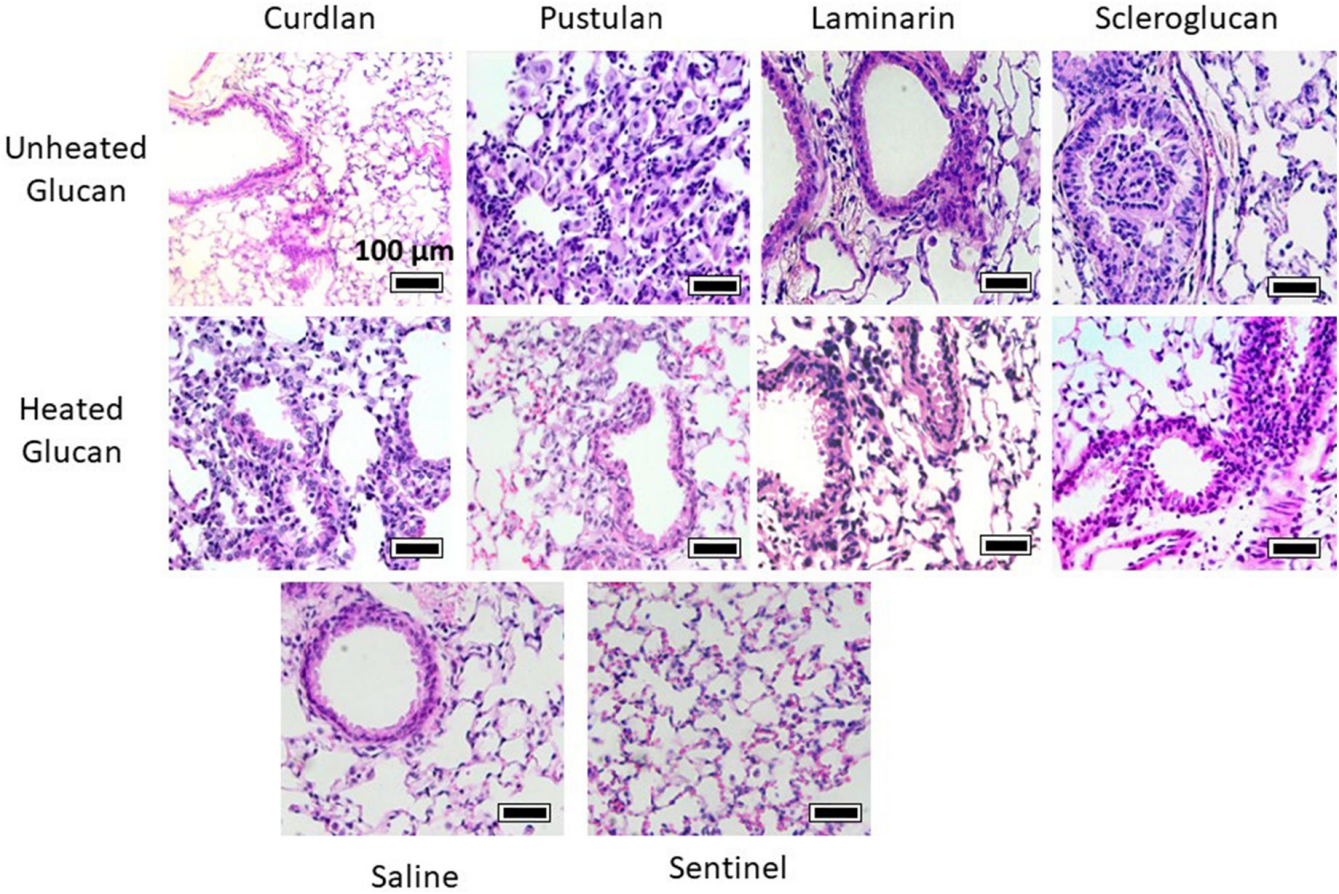
Histopathology of BDG‐exposed lungs. Mice were exposed to unheated or heated glucan compounds and lungs were stained with hematoxylin and eosin stain; representative photomicrographs selected from separate experiments representing similar results per condition. All scale bars = 100 μm.

Next, we analyzed lung histology in animals exposed to BDG with or without heating. Overall, glucans induced neutrophil and lymphocyte migration to the airspace. Pustulan induced the greatest visible infiltrate, consistent with Figure 1, but this was not extensively quantified. Heating of insoluble, linear curdlan increased inflammatory infiltrate while unheated glucans otherwise had the greatest effect, Figure 6.

### 3.4 Response of left lung lobe primary cells to secondary stimuli

We next tested whether primary sensitization to BDGs affected ex‐vivo lung cell cytokine production upon secondary stimuli. Lobes from the left lung were dissected, repeatedly washed, and cell suspension plated in triplicate alongside vehicle, BDG, LPS, or CD3 mAb (to stimulate T‐cells). Cell supernatant was collected after 48 h, and cytokines assessed. Cell viability was quantified using trypan blue stain and determined to be >90%.

**FIGURE 7:**
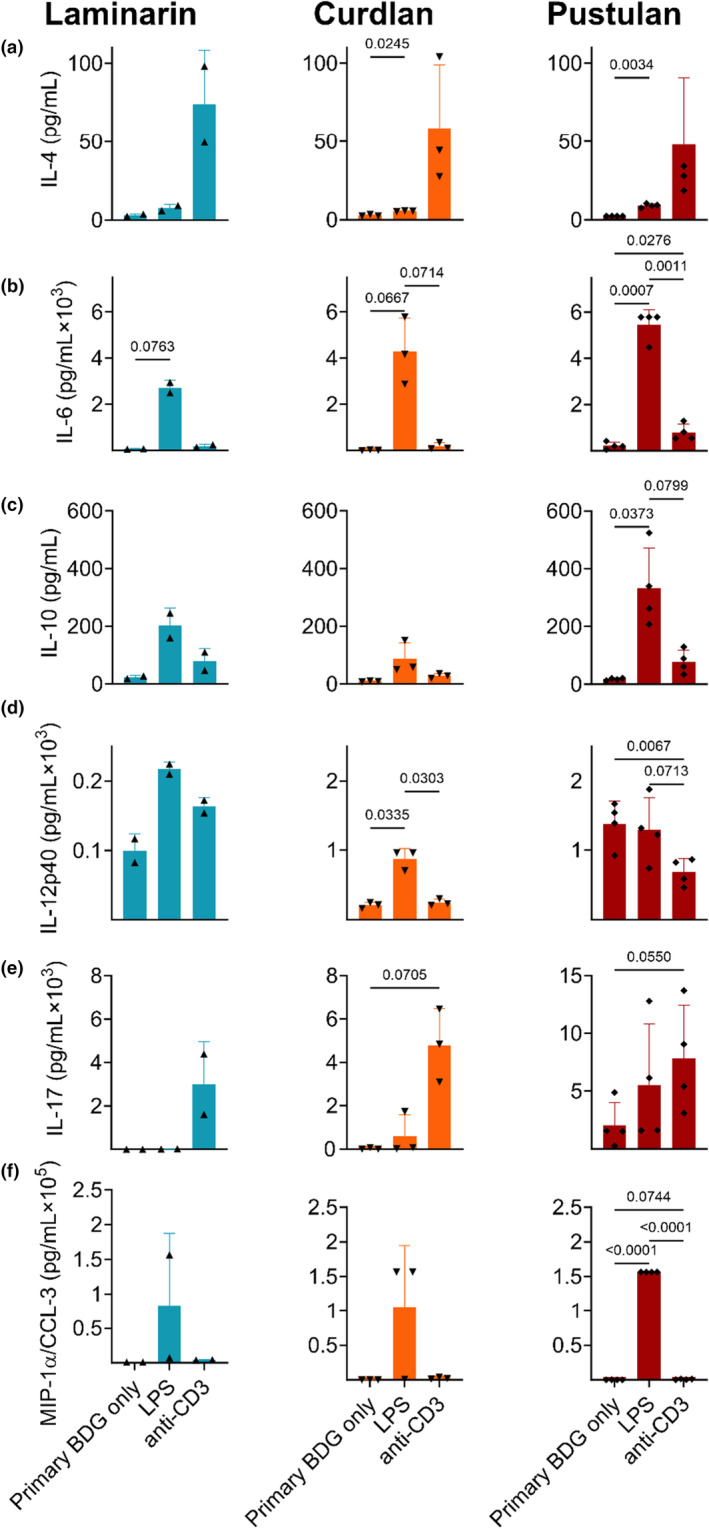
Effect of secondary stimuli on excised primary cells removed from lung tissue and plated at the monolayer. Effect of LPS and anti‐CD3 antibody on (a) IL‐4, (b) IL‐6, (c) IL‐10 D. IL‐12p40, (e) IL‐17, (f) MIP‐1α/CCL‐3 concentrations in cell supernatant; significance determined by repeated measures one‐way ANOVA with Tukey's multiple comparisons test; *n* = 2–4, performed in triplicate.

Lipopolysaccharide (LPS) exposure to cells previously sensitized (in vivo) increased IL‐4 (1° stimuli: curdlan, pustulan), IL‐6 (all), IL‐10 (pustulan), IL‐12p40 (curdlan), and MIP‐1α/CCL‐3 (pustulan) cytokine concentrations (Figure 7a–d,f). We next assessed T‐cell‐specific cytokine induction by addition of a CD3 antibody, observing differential effects based on initial BDG stimulation. For example, compared to baseline, IL‐4 increased across all conditions, however not significantly; IL‐6 increased (1° stimulus: pustulan), IL‐12p40 decreased (pustulan), IL‐17 increased (all trend), and MIP‐1α/CCL‐3 increased (pustulan trend) (Figure 7). Secondary exposure to BDGs did not increase cytokine expression, except in the case of curdlan, which increased IL‐4, IL‐6, IL‐10, IL12p40, and MIP‐1α/CCL‐3 production compared to control (Figure S6).

## 4 DISCUSSION

We tested the effect of four structurally distinct β‐d‐glucans on murine immune response, finding that soluble (1 → 6)‐linear BDG pustulan exposure led to the most pronounced pro‐inflammatory effects. Pustulan is from lichen; exposure to which likely occurs in occupations working with wood dust and in the lumber/forestry industry, which increase the risk of pulmonary fibrosis and sarcoidosis (Blanc et al., 2019; Cummings et al., 1959). Farmers are potentially exposed to scleroglucan when harvesting soybeans (Roy & Thorne, 2003). Curdlan originates from environmental bacteria, making its exposure common. Laminarin originates from algae, making exposure potentially less likely.

Pustulan increased total BAL protein concentration, immune cell infiltrate, and cytokine concentrations, IgE and IgG2a, compared to control Figures 1, 2, 3, 4, 6. The neutrophil and lymphocyte migration into the airspace observed in lung micrographs was reminiscent of a murine asthma model (Xi et al., 2021); however, our cytokine data supports a mixed Th1/17 immune response to BDGs (Figure 3). Gene–environment interactions likely lead to distinct mold‐induced phenotypes (Barnes, 1999). For example, sarcoidosis, which is typically considered a Th1 and/or Th17‐dominate disease (Facco et al., 2011; Katchar et al., 2003), risk is elevated in workers exposed to a water‐damaged building, yet this same building also led to increased asthma incidence and symptoms (Laney et al., 2009).

Allergic asthma is an inflammatory lung disease characterized by eosinophil infiltrate and bronchial hyperreactivity (Adamko et al., 2003; Galli et al., 2000). Highly branched scleroglucan (1 → 3, 1 → 6)‐β‐d‐glucan exposure in asthmatic children has been shown to decrease FEV_1_ and greatly increases the odds of emergency visits for asthma (Blatter et al., 2014). In this work, scleroglucan induced certain hallmarks of asthma, including increased concentration of macrophages in the airspace (Figure 1b), and serum IgE (Figure 2a) (van der Veen et al., 2020). However, Th1 IgG2a is also increased (Figure 2b).

While allergic disease is predominated by T cell differentiation into Th2 cell response, Th1 cytokine, IFN‐γ, can also potentiate lung injury induced by Th2 cytokine IL‐13 (Ford et al., 2001). Interplay between Th1 and Th2 cytokines can result in allergic disease pathology. Surprisingly, pustulan also reduced Th2 cytokine IL‐9 (Chen et al., 2019) and suppressor IL‐10 concentrations in BAL, Figure 3, suggesting a change from Th2 towards Th1 or Th17. While we did not observe significant differences in IFN‐γ or TNF‐α production after exposures, Figure S5, curdlan and pustulan tended to increase IFN‐γ concentrations (both *p* < 0.19). However, a lack of IFN‐γ response is consistent with findings from (Hadebe et al., 2016), who found IFN‐γ did not respond to a co‐BDG + house dust mite allergen model.

We performed a sub‐study to evaluate the effect of glucan solubility on lung inflammation given BDGs present in different conformations, which may alter a host's physiological response. While autoclaved BDGs reduced inflammatory infiltrate into BAL (Figure 1), glucan solubility often increased cytokine concentrations in BAL (Figure 5). Soluble glucans scleroglucan and pustulan increased systemic inflammatory markers (IgE and IgG2a; Figure 2). Increased serum IgG has been observed in a small cohort of patients with pulmonary fibrosis (Komura et al., 2008). Scleroglucan and pustulan also increased BAL cell counts and macrophage concentrations. Macrophages are increasingly recognized for their role in development of pulmonary fibrosis (Bhattacharyya et al., 2022; Laskin et al., 2019; Wynn & Vannella, 2016) with excessive M2 macrophages playing a pro‐fibrotic role (Lis‐López et al., 2021). Macrophage response to BDGs has been demonstrated to be independent of the classic BDG receptors Dectin‐1 and TLR2 (Kelly et al., 2008; Smeekens et al., 2015), because Dectin‐1 is not independently responsible for Th2 sensitization in a BDG (+house dust mite) mouse model (Hadebe et al., 2016). Pustulan also significantly increased IL‐17 concentrations in the airspace (Figure 3) which is thought to play a direct role in lung fibrosis (Wilson et al., 2010; Zhang et al., 2019). Dectin‐1 plays a protective role in fibrosis by suppressing TLR4 activation (Seifert et al., 2015). Dectin‐1 signaling is effectively halted when soluble BDGs are detected by innate immune cells (Goodridge et al., 2011; Seifert et al., 2015). The absence of Dectin‐1 signaling upon exposure to soluble glucans likely increases the role of other BDG recognition components of the plasma membrane, such as TLR4 and lactosylceramide (Brown, 2006; Sahasrabudhe et al., 2016). The presence of IL‐17, observed in BAL of the pustulan‐exposure group, Figure 3, increases TLR4 expression (Tang et al., 2010). TLR4 activation is required for IL‐17 induced effects, including neutrophil infiltrate (Tang et al., 2010). Abrogation of Dectin‐1 signaling may lead to lung fibrosis after exposure to soluble glucans. Future work to determine the role of BDG exposure and lung fibrosis is warranted.

We analyzed the effect of secondary stimulation (endotoxin, anti‐CD3, BDG) in plated lung cells immediately following sacrifice to assess in vitro effects of exposure. Because these cells were stimulated immediately following the wash step and not after prolonged differentiation, this allowed for T‐cell‐specific cytokine response, which we confirmed by the observation that IL‐4 and IL‐17 increased after CD3 stimulation (Figure 7). IL‐17 is known to protect against fungal and bacterial infection through neutrophil recruitment, increased antimicrobial peptide production, and improved barrier protection. This is likely due to increased T‐cell response, which is the primary producer of IL‐17; however, the effect is not limited to T‐cell‐specific response given its can be produced by cytokines (IL‐1β, IL‐23), innate lymphoid cells, natural killer cells, and mast cells (Mills, 2023). Because IL‐4 also tended to increase under these conditions, it is possible mast cells also contribute to the increased cytokine production observed with the CD3 antibody (McLeod et al., 2015).

Isolated lung cells that were exposed to secondary stimulation (LPS, anti‐CD3, secondary BDG exposure) had exaggerated cytokine response to LPS, indicating the importance of BDG priming in TLR4‐mediated (Lu et al., 2008) inflammatory response, Figure 7. BDG exposure is known to enhance immune response and protect against bacterial infection (Stothers et al., 2021), likely partially explaining the excessive cytokine release observed after stimuli. Secondary BDG stimulation of left lung cells did not enhance baseline cytokine response except in the case of curdlan (Figure S6) which significantly increased all measured cytokines and chemokines except IL‐17. It especially increased MIP1a/CCL‐3, which hones dendritic cells to drain lymph nodes and is important in lung diseases, including sarcoidosis (Bhavsar et al., 2015; Schaller et al., 2017). In most experiments, pustulan induced the greatest inflammatory response; however, secondary curdlan exposure increased all cytokine expression in isolated lung cells in vitro except IL‐17 (Figure S6). This may indicate lung‐specific sensitization to curdlan. Further, the timepoint of BAL collection (31 days post initial exposure) may not have captured the peak inflammatory response to individual BDGs.

Study strengths include the systematic use of characterized unique BDGs in an animal model of inflammation and the identification of one extremely pro‐inflammatory BDG (pustulan). This study has several limitations. Cytokines were only measured in the lung compartment and not systemically; responses in murine lungs may not equate to human responses, and BAL macrophage lineage was not assessed. While we observed differences in response to autoclaved BDGs in BAL (decreased inflammatory infiltrate, typically more cytokines), the sample size of this sub‐study was smaller, making definitive conclusions harder to draw. Lastly, certain cell‐types better survive mechanical disruption, and each cell‐type has unique adherent properties; therefore, left lung cells plated at the monolayer were likely comprised of various cell types, including those found in the BAL differential count, Figure 1. While we did not quantify cell‐types, cell isolation was performed identically in all study groups, and we observed a response to secondary LPS and anti‐CD3 simulation, indicating lung cells were primed to respond to antigen challenge.

Overall, we observed increased inflammatory infiltrate, immune cells, and a strong Th1 and Th17 BAL response to BDG exposure, yet serum IgE was elevated, indicative of Th2 allergen response. Secondary stimulation (LPS or BDG) of glucan‐primed lung cells magnified the inflammatory response, especially to secondary curdlan stimulation, demonstrating the importance of prior BDG immune priming. Further, this work demonstrated structural differences in BDGs result in unique immunological responses in murine lungs. Exposures to fungi should be considered in diverse lung disease phenotypes.

## FUNDING INFORMATION

NIH P30 ES005605, University of Iowa Center for Health Effects of Environmental Contamination (CHEEC), Foundation for Sarcoidosis Research Pilot Grant.

## CONFLICT OF INTEREST STATEMENT

All authors declare no conflicts of interest.

## ETHICS STATEMENT

This research did not require IRB approval because it did not involve human studies.

## Supporting information


Data S1.

## Data Availability

Data generated during the current study are available from the corresponding author upon request.
