# Supplementary material for: Exposure to structurally unique β‐d‐glucans differentially affects inflammatory responses in male mouse lungs
Source: Physiol Rep. 2024 Jun 23;12(12):e16115. doi: 10.14814/phy2.16115 (PMC11194181; doi:10.14814/phy2.16115)
Supplement: Supplementary file 1 — Data S1. [file PHY2-12-e16115-s001.docx]

**MATERIALS AND METHODS**

*Reagents and catalogue numbers:*

Laminarin ((1→3) (1→6)-branched BDG; Sigma-Aldrich, Inc., St. Louis, MO); cat# L9634
Curdlan (1→3)-linear BDG; Wako, Richmond, VA; cat# NC0353129
Pustulan ((1→6)-linear BDG; Calbiochem Inc., La Jolla, CA); cat# YP15423
Kinetic chromogenic Limulus amebocyte lysate assay (Lonza, USA); product number 50-650H
NaIO4 (Fisher scientific company); cat# 60045816
Ethylene glycol (Fisher scientific company); cat# NC2316125
Carbohydrate concentration kit instructions (Thermo Fisher Scientific, Waltham, MA); cat# 501098805
Bovine Serum Albumin (Sigma Aldrich); cat# A3803-50G
Phenol (Fisher Scientific); cat# NC1409795
Sulfuric acid (Fisher scientific company); cat# 60046055
Na cyanoborohydride (Fisher Scientific); cat# NC2333889
Centriplus concentrator (Thermo Fisher Scientific); cat# 88523
Trifluoroacetic acid (CF₃CO₂H) (Fisher scientific company); cat# P128902
Histology performed in pathology facilities, Formaldhyde, cat# 60-044-881
IL-6, IL-9, IL-10, IL-12p40, IL-17, CXCL-1, MCP-1/CCL-2, MIP-1α/CCL-3, MIP-1β/CCL-4Eotaxin (Bio-Rad); cat#17010263
Sandwich enzyme-linked immunoassays (ELISAs) (eBioscience, Invitrogen, Waltham, MA) for IFN-γ (cat#BDB551866), TGF-β (cat#501129049) and TNF-α (cat#BMS607-3)
Hank’s Balanced Salt Solution media with phenol red (Fisher Scientific); cat#NC1100320
Diff Quick Stain Set (Thermo Scientific, USA); cat# NC0851891
QuantiPro BCA assay kit (kit included BSA) (Sigma-Aldrich) ; cat# QPBCA
RPMI 1640 (GIBCO, Grand Island, NY); cat#31870017
100 µm nylon cell strainer (BD Labware, Franklin, NJ); cat#07-201-432
Fetal Calf Serum (FCS) (Sigma Aldrich); cat#C8056
HEPES buffer (Sigma Aldrich); cat# 137264
L-Glutamine (Sigma Aldrich); Cat# 59202C
β-Mercaptoethanol (Sigma Aldrich); cat#63689
Sodium Pyruvate (Fisher Scientific); cat#116-079-721
100U/ml penicillin-100 mg/ml streptomycin (GIBCO); cat# 15140163
Trypan blue stain (Fisher Scientific); cat#15250061
96-well microtiter plates (Corning, Cambridge, MA); cat#CLS353077
*Escherichia coli* 0111:B4 (Sigma-Aldrich); cat#501489343
anti-CD3 mAb (clone #2C11, ATCC); cat# ATCC CRL-1975, see PMID: 33067820
IL-4, IL-6, IL-10, IL-12/23p40, IL-17 and MIP-1α by multiplex immunoassay (Bio-Rad, Hercules, CA); cat#17010264
Total IgE sandwich ELISA (Invitrogen, Waltham, MA); cat# BDB2555248
Total IgG2a sandwich ELISA (Invitrogen, Waltham, MA); cat# BDB552576


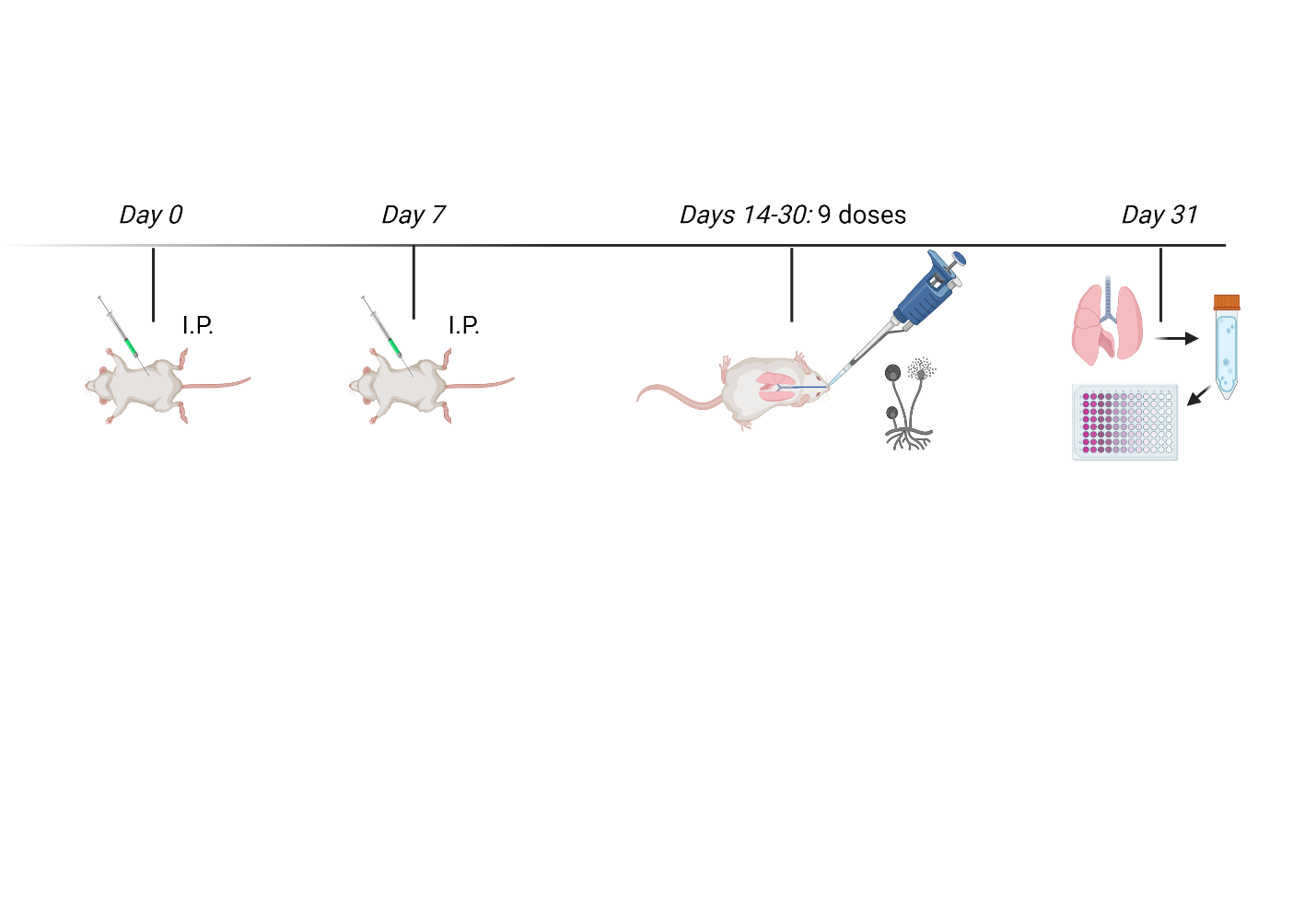


**Figure S1. Schematic of study design protocol.** Mice received intraperitoneal injections of BSA-glucan conjugates at days 0 and 7, while negative control mice were exposed to saline. On days 14-16, 21-23, and 28-30, mice were exposed to BDGs (25 µg BDG/mouse) intranasally, while control mice received saline solution. Harvest was performed on day 31, at which point cytokin; created with BioRender.com.

**
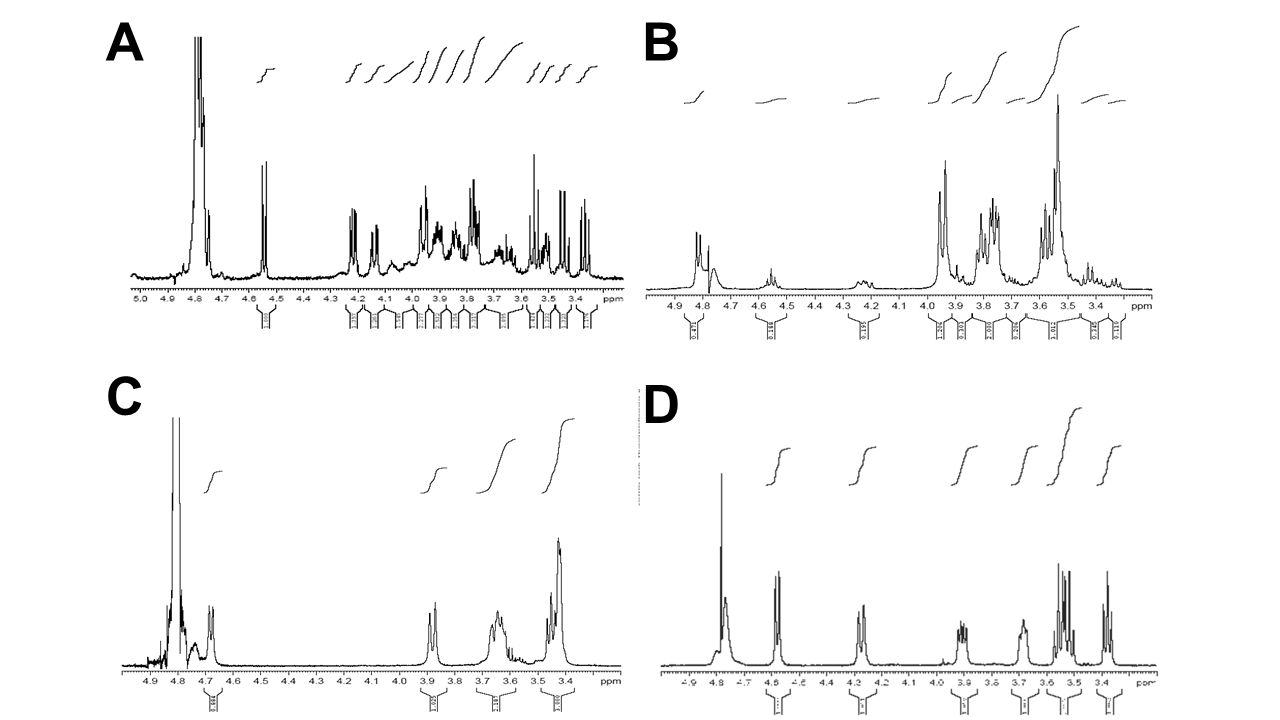
Figure S2. Glucan compound structure assessed by nuclear magnetic resonance imaging.** Glucans were suspended in deuterium oxide and assessed by 1H NMR spectra; **A.** Scleroglucan **B.** Laminarin **C.** Curdlan, and **D.** Pustulan.

**
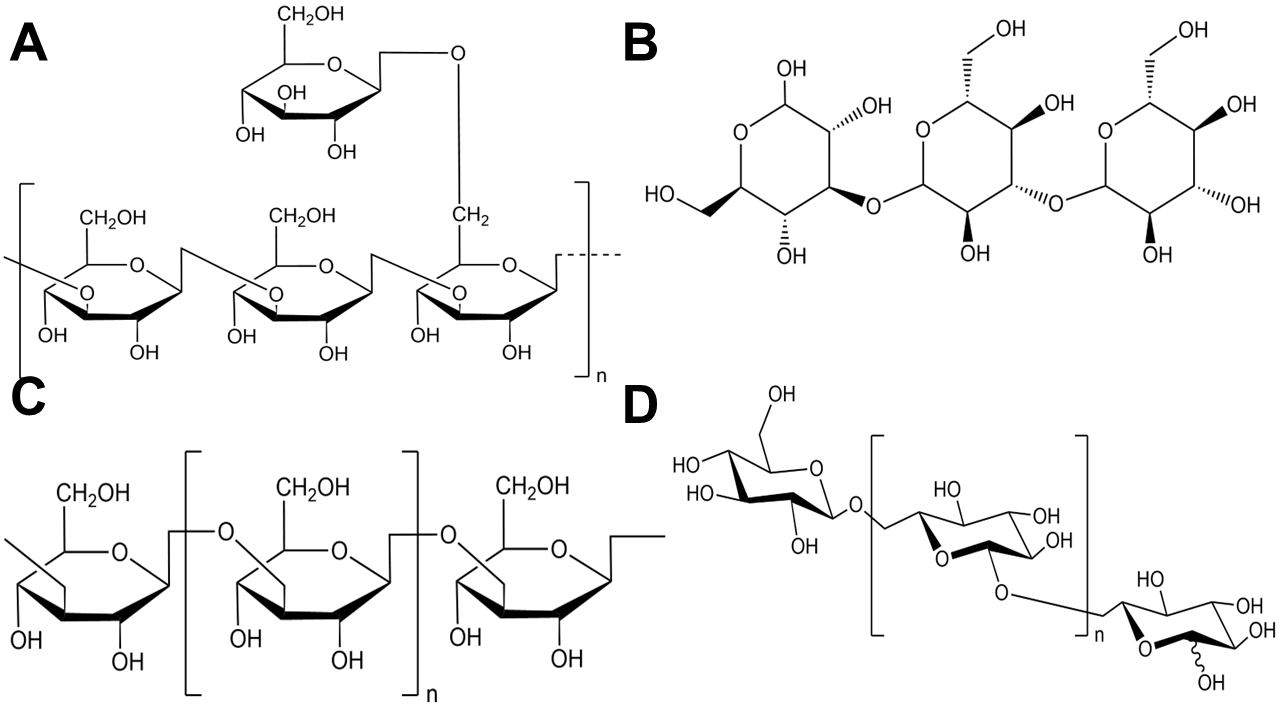
Figure S3. A.** Scleroglucan (1→3) (1→6)-highly branched BDG. **B.** Laminarin ((1→3) (1→6)-branched BDG. **C.** Curdlan (1→3)-linear BDG. **D.** Pustulan (1→6)-linear BDG.

**
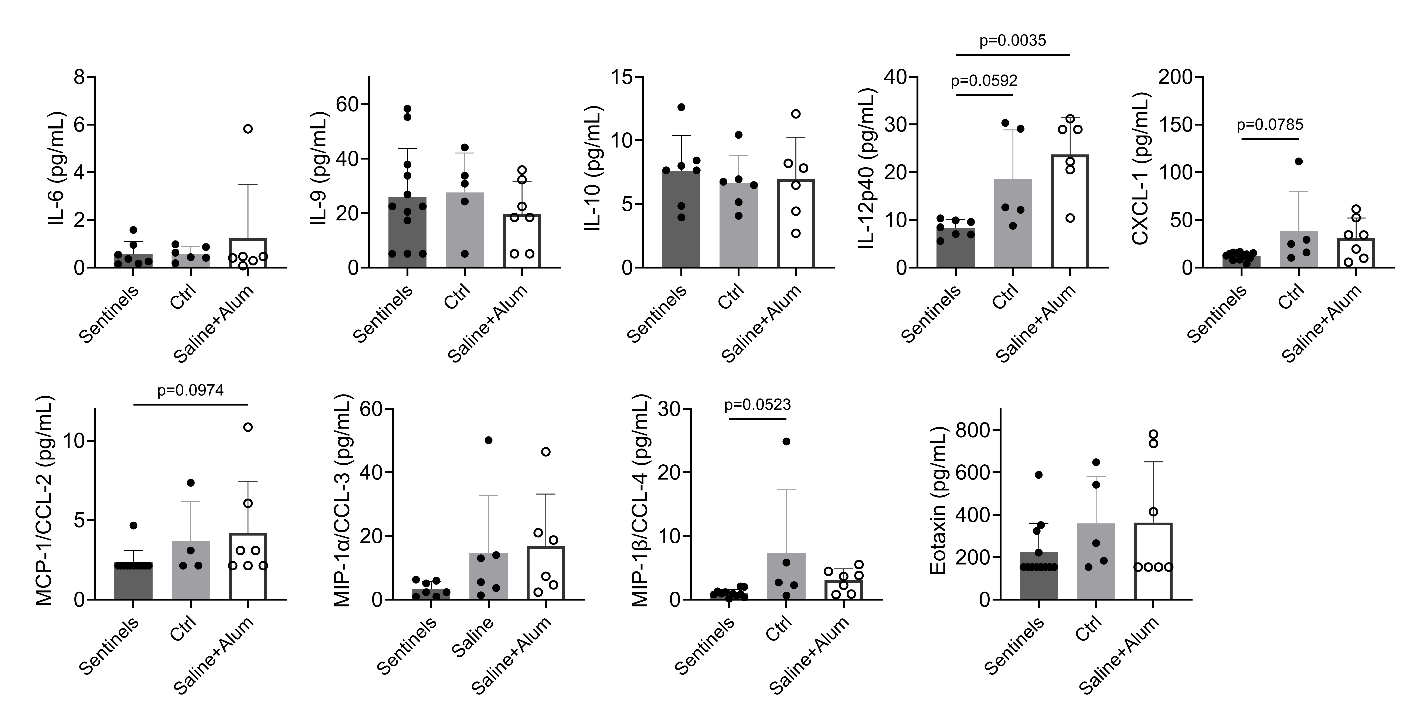
Figure S4. Comparisons of cytokine production between control conditions.** Bronchoalveolar lavage cytokine production was similar between sentinels, saline, and saline+alum conditions, with no significant differences observed between saline or saline+alum exposed animals. IL-17 and IL-4 data are not shown as concentrations were largely below the limit of detection. Ordinary one-way ANOVA or Kruskal-Wallis tests were performed based on Kolmogorov-Smirnov normality test results, *p* values <0.1 displayed.


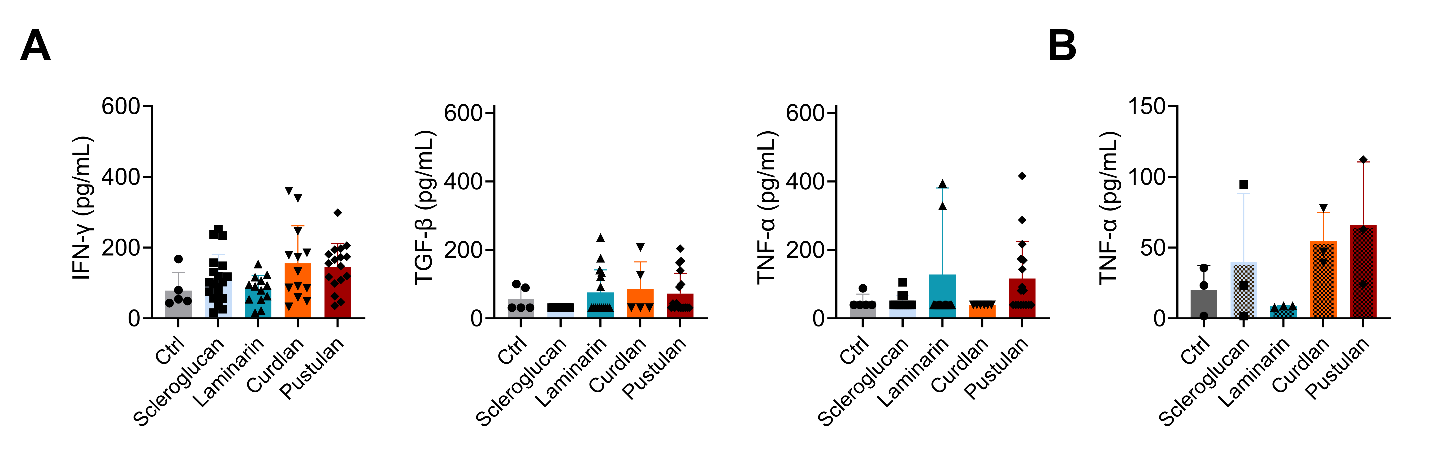


**Figure S5. Cytokines identified by ELISA. A.** No significant differences were observed between IFN-γ, TGF-β and TNF-α vs. saline controls, measured by ELISA in BAL after exposure to unheated glucans. **B.** No significant differences were observed in TNF-α vs. sentinel controls, measured by ELISA after mice were exposed to solubilized (heated) glucans in sub-study; ordinary one-way ANOVA or Kruskal-Wallis tests were performed based on Kolmogorov-Smirnov normality test results all *p*>0.1.

**
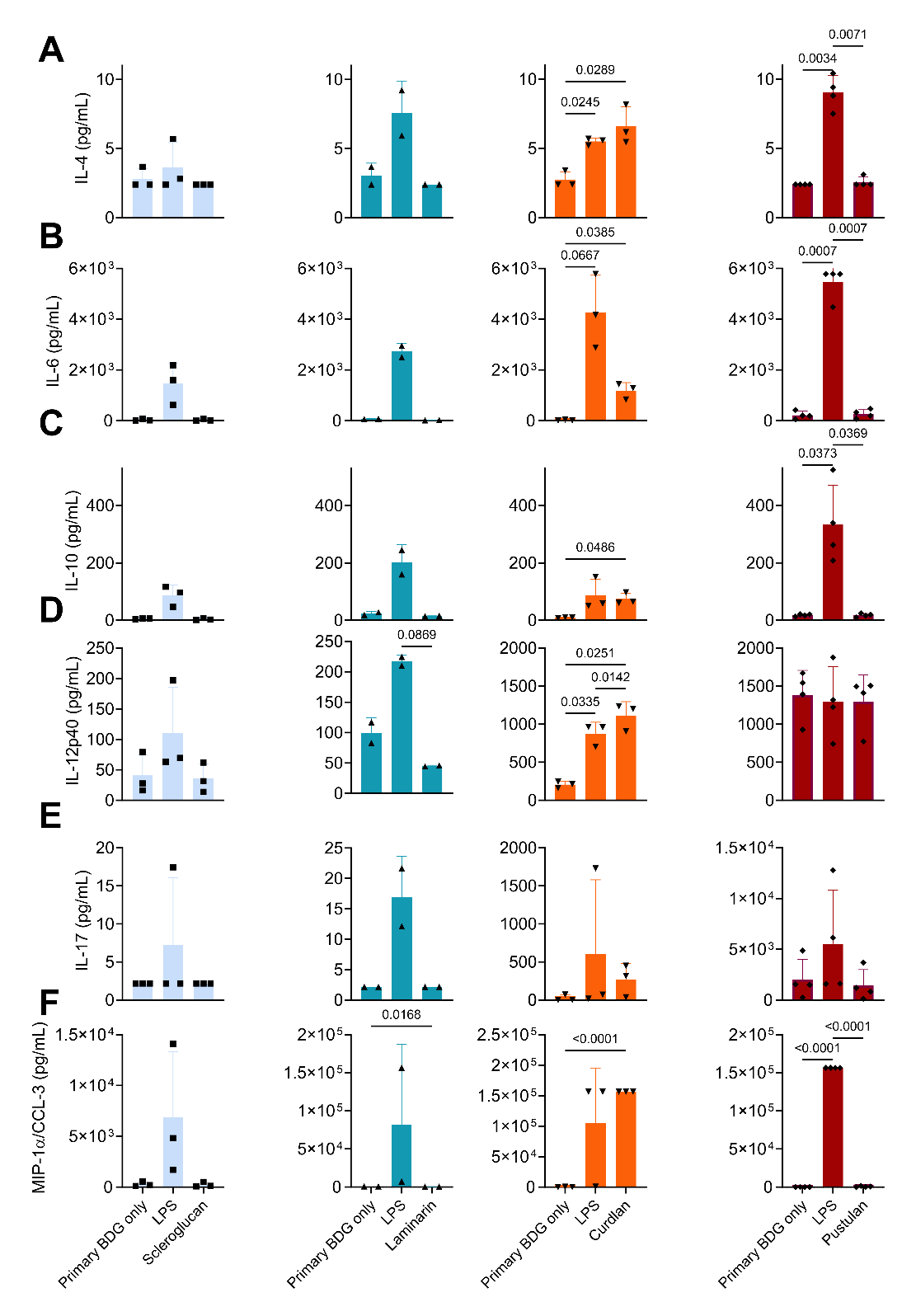
**

**Figure S6. Secondary curdlan stimulation in isolated lung cells results in increased cytokine expression. A.** Effect of secondary scleroglucan, laminarin, curdlan or pustulan stimulation on IL-4 production by isolated lung cells. **B.** Effect of secondary BDG stimulation on IL-6 production by isolated lung cells. **C.** Effect of secondary BDG stimulation on IL-10 production by isolated lung cells. **D.** Effect of secondary BDG stimulation on IL-12p40 production by isolated lung cells. **E.** Effect of secondary BDG stimulation on IL-17 production by isolated lung cells. **F.** Effect of secondary BDG stimulation on MIP-1α/CCL-3 by isolated lung cells; significance determined by repeated measures one-way ANOVA with Tukey’s multiple comparisons test; *p*<0.10 shown.
